# Editorial: Cardiac reverse remodeling after novel heart failure therapies

**DOI:** 10.3389/fcvm.2024.1362760

**Published:** 2024-02-07

**Authors:** Massimo Mapelli, Elisabetta Salvioni, Ofer Havakuk, Piergiuseppe Agostoni

**Affiliations:** ^1^ Centro Cardiologico Monzino, Istituto di Ricovero e Cura a Carattere Scientifico (IRCCS), Milan, Italy; ^2^ Department of Clinical Sciences and Community Health, Cardiovascular Section, University of Milan, Milan, Italy; ^3^ Cardiology Division, Tel Aviv Sourasky Medical Center, Tel Aviv University, Tel Aviv, Israel

**Keywords:** heart failure, heart failure drug therapy, device therapies, heart failure prognosis, reverse remodeling in heart failure

**Editorial on the Research Topic** Cardiac reverse remodeling after novel heart failure therapies

In the realm of cardiovascular research, the focus has increasingly shifted towards unraveling the complexities of heart failure (HF)—a syndrome once viewed as relentlessly progressive. More than a decade ago, Gheorghiade et al. (1) painted a somber picture, describing HF as a highly progressive, chronic syndrome, marked by an inexorable decline in both quality of life and prognosis, accompanied by an increase in mortality. In the context of this syndrome, once cast a shadow of inevitability, a new dawn emerges—a dawn marked by innovation and multidisciplinary approach. As we traverse the pages of these five groundbreaking studies (Sacharczuk et al., Yokoyama et al., Zhang et al., Yan and Grazette, Dong et al.), each contributing a unique angle to the evolving narrative of cardiac reverse remodeling, we can proudly recognize how the trajectories of our patients can be diverted from what was previously perceived as a fate of inexorable worsening, embarking a journey against the tide of HF progression Beyond the scientific insights, a common thread emerges—underscoring the pivotal and varied role played by specialists in HF.

Our journey commences with Yokoyama et al. retrospective exploration into transcatheter mitral valve repair and the potential of combination therapy of different transcatheter techniques to treat mitral valve disease (COMBO-TMVr) in high-risk patients with symptomatic severe mitral regurgitation. The study focused on combining mitral transcatheter edge-to-edge repair with other devices targeting different components of the mitral valve apparatus. The COMBO-TMVr strategy demonstrated a sustained significant reverse remodeling of the overloaded left cardiac chambers at 1 month and 1 year of follow-up, leading to a reduction in left ventricular dimensions, volumes, and mass.

As shown by Adamo et al. (2) the role of percutaneous mitral valve repair has evolved rapidly in the last years, thanks to the technical innovations with new devices. This small, observational, single-center study not only suggests a concomitant approach as a possible strategy for complex mitral valve disease, but highlights how a COMBO-TMVr strategy can led to a significant reduction in heart chambers volumes.

Continuing forward, Sacharczuk et al. guide us through the intricate landscape of HF treatment. Here, the HF specialist emerges as the linchpin, translating personalized approaches into effective interventions, ensuring a seamless integration of therapies aimed at improving cardiac function, as also demonstrated in both small and larger trial (3–6).

Our third exploration (Zhang et al.) investigates the clinical performance and safety of cardiac conduction system pacing (CSP), specifically His-bundle pacing (HBP) and left branch bundle pacing (LBBP), in patients with HF and mildly reduced ejection fraction (HFmrEF) with a high percentage of ventricular pacing. The study demonstrates that CSP, particularly LBBP, improves clinical outcomes in these patients, with a high success rate and stable pacing thresholds. The results suggest that CSP could be a preferable option for HFmrEF patients with ventricular pacing dependence, emphasizing the potential benefits of LBBP, especially in cases of infranodal atrioventricular block.

From a different point of view, Yan and Grazette further emphasize the HF specialist's role in deciphering the intricate web of cardiac remodeling with a comprehensive review that unravels the predictive role of biomarkers and imaging monitoring, the article discusses the diverse clinical presentations and outcomes in HF, underlying the importance of predicting HF recovery for guiding clinical practice. It explores various biomarkers, including NT-proBNP, cardiac troponins, soluble ST2, and galectin-3, assessing their potential in predicting structural, functional, and clinical recovery in HF patients. The review also touches on the role of serial echocardiography and cardiac magnetic resonance imaging in predicting HF improvement. The identification of reliable predictors for recovery holds significance for prognosis estimation, therapeutic evaluation, and personalized patient care.

Our final exploration, led by Dong et al., introduces the innovative loop technique, demonstrating its safety and effectiveness in preventing left ventricle lead dislocation, leading to favorable therapeutic outcomes. The study highlights the importance of innovative approaches like the loop technique to enhance the success of cardiac resynchronization therapy, providing a minimally invasive solution without additional costs. In this scenario, the HF specialist stands as the mediator, ensuring the seamless integration of device-related interventions into the broader strategy for HF management.

As we conclude this journey through transformative studies, we acknowledge the crucial role of the HF specialist—a central figure navigating the complexities of this multifaceted syndrome. In the intricate dance of pharmacological breakthroughs, electrical interventions, percutaneous valves, and laboratory biomarkers, the HF specialist emerges as the protagonist, weaving together the threads of diverse specialties into a cohesive strategy for each patient. Looking ahead, the future landscape must not only involve assessments of patients at rest but also under exercise conditions (functional evaluation) (7, 8). In this context, it becomes imperative to enhance our ability to delineate the various potential trajectories of HF progression in a given individual, adopting an increasingly personalized diagnostic and therapeutic approach. Therefore, a longitudinal monitoring is crucial to better understand the evolution of the HF syndrome and determine the correct time intervals for early identification of patient deterioration.

**Figure 1:**
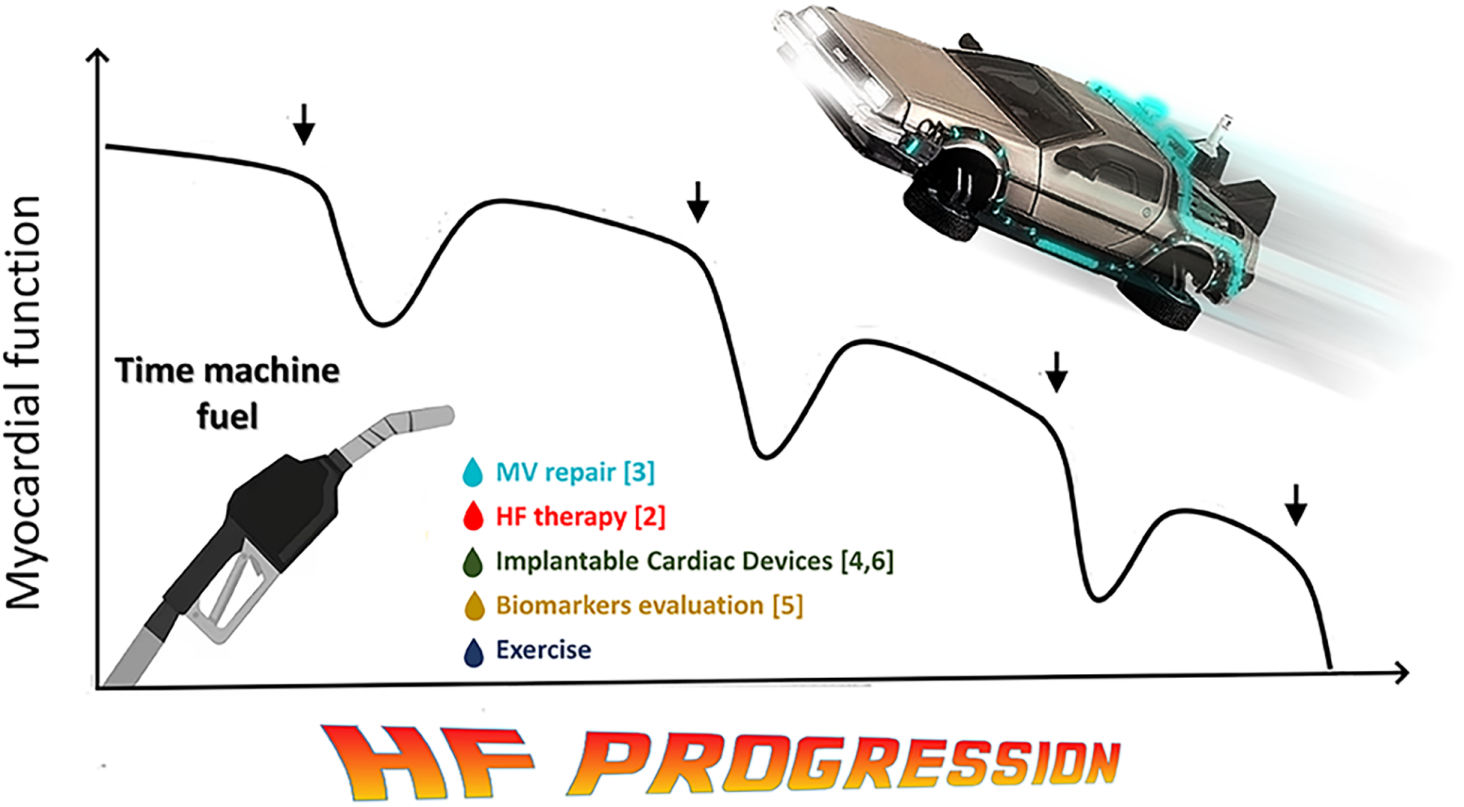
Back to the future of HF. Taking a cue from Gheorghiade's famous now-historical image illustrating the progression of HF syndrome (1), this figure hypothesizes that the diagnostic and therapeutic tools currently available can try to reverse the natural worsening of the disease and, as in a time machine, bring the heart back to less advanced stages of disease, prolonging survival and improving patients’ quality of life. HF: heart failure.

Understanding and discerning the possible interventions to alter these trajectories, steering a patient from an unfavorable course to a more favorable one, are pivotal. In this scenario, the HF expert becomes the bridge between specialties, ensuring a unified approach and a steadfast strategy for patients facing the challenges of cardiac reverse remodeling. In a cinematic metaphor (9), he embodies the protagonist—a guiding force in a plot where collaboration, innovation, and hope intertwine to invert the relentless HF progression as the pilot of a time machine (Figure 1).
